# Echinatin inhibits tumor growth and synergizes with chemotherapeutic agents against human bladder cancer cells by activating p38 and suppressing Wnt/β-catenin pathways

**DOI:** 10.1016/j.gendis.2023.03.031

**Published:** 2023-05-18

**Authors:** Xiaoxuan Wang, Lijuan Luo, Jingtao Xu, Qiuping Lu, Haichao Xia, Yanran Huang, Lulu Zhang, Liping Xie, Habu Jiwa, Shiqiong Liang, Xiaoji Luo, Jinyong Luo

**Affiliations:** a Key Laboratory of Diagnostic Medicine Designated By the Chinese Ministry of Education, School of Laboratory Medicine, Chongqing Medical University, Chongqing 400016, China; b Department of Orthopedics, The First Affiliated Hospital of Chongqing Medical University, Chongqing 400042, China

**Keywords:** Bladder cancer, Combination chemotherapy, Echinatin, p38, Wnt/β-catenin

## Abstract

Bladder cancer (BC) is one of the most common malignant tumors in the urinary system. Due to the poor prognosis and high mortality rate of the disease, it is urgent to develop new drugs with high efficacy and low toxicity to treat BC. Echinatin (Ecn) is a bioactive natural flavonoid oflicorice that has attracted special attention for its promising anti-tumor potential. Herein, we explored the inhibitory effects of Echinatin on BC cells and probed the possible molecular mechanism. We found that Ecn*in vitro* inhibited the proliferation, migration, and invasion, arrested the cell cycle at the G2/M phase, and promoted apoptosis in BC cells. Besides, Ecn had no notable cytotoxicity towards human normal cells. We subsequently confirmed that Ecn restrained xenograft tumor growth and metastasis of BC cells *in vivo*. Mechanistically, Ecn activated the p38 signaling pathway but inactivated the Wnt/β-catenin signaling pathway, while over-expression of β-catenin and the p38 inhibitor both attenuated the inhibitory effects of Ecn on BC cells. Remarkably, Ecn combined with cisplatin (DDP) or gemcitabine (Gem) had synergistic inhibitory effects on BC cells. In summary, our results validate that Ecn inhibits the tumor growth of human BC cells via p38 and Wnt/β-catenin signaling pathways. More meaningfully, our results suggest a potential strategy to enhance DDP- or Gem-induced inhibitory effects on BC cells by combining with Ecn.

## Introduction

Bladder cancer (BC) is a malignant tumor that originates from the bladder mucous membrane and represents one of the most lethal malignancies involving the genitourinary tract.^1^ The World Health Organization classifies bladder cancer as the 10th most frequently diagnosed cancer worldwide. Data from GLOBOCAN2020 reported that over 550,000 new BC cases were diagnosed yearly, resulting in greater than 200,000 deaths each year.^2^^,^^3^ Currently, the guideline-recommended treatment methods for BC include tumor section, bladder perfusion therapy, systemic chemotherapy and radiation therapy.^4^ Even so, the overall 5-year survival rate of BC patients remains at a low level and the risk of tumor recurrence is still very high after surgery.^5^ BC is prone to metastasis, and about 25% of BC are found to invade muscles with distant metastases when the diagnosis is confirmed. Metastatic BC patients face a worse prognosis and the 5-year survival rate is merely 5%.^6^, ^7^, ^8^, ^9^ Clinically, the mainstay of treatment for patients with metastatic BC is systemic chemotherapy. The current standard chemotherapy regimen for BC is a combination of gemcitabine and cisplatin (GC therapy). However, the median overall survival time of patients treated with GC is only 14 months.^10^^,^^11^ Besides, non-selective cytotoxicity and acquired resistance after chemotherapy drug application remain the predominant causes of therapeutic failure.^12^ Hence, developing less toxic and more effective therapeutic drug candidates is desperately needed.

Recently, traditional Chinese herbs with anti-tumor activity have drawn rising attention by virtue of their safety and efficacy.^13^ Aside from the direct tumor-killing activity, Chinese herbs can also increase the sensitivity of tumor cells to chemotherapy and potentiate the tumoricidal effect of chemotherapeutic drugs.^14^ Licorice is a traditional Chinese herbal medicine with nutritional and therapeutic values and has been extensively employed in China for around 2000 years. Nowadays, licorice and its extract ingredients have been approved by China Food and Drug Administration as dietary supplements and medicines.^15^ Licorice and its products are also widely used in other Asian countries such as Japan and South Korea, and are sold worldwide.^16^ Licorice possesses a wide range of pharmacological actions, including antioxidant, anti-ulcer, anti-inflammatory, and anti-carcinogenic activities.^17^^,^^18^ Echinatin (Ecn) is an active flavonoid compound extracted from the roots and rhizomes of licorice, which has been widely utilized as a natural sweetener and food additive, as well as in traditional Chinese medicine.^19^^,^^20^ Numerous studies have revealed that Ecn has various pharmacological actions such as anti-oxidation, anti-inflammation, anti-parasitism, and cardio-protection.^21^^,^^22^ Moreover, Ecn exerted anti-tumor activity against esophageal cancer cells, lung cancer cells, and colorectal cancer cells by inducing autophagy and/or apoptosis.^23^, ^24^, ^25^ However, more experiments are needed to verify whether Ecn is really a pan-anticancer drug and to delineate the mechanism involved.

In this study, we sought to evaluate the anti-tumor effects of Ecn on BC cells and to probe its specific molecular mechanism. We demonstrated that Ecn inhibited the proliferation, migration, and invasion, whereas promoted the apoptosis of BC cells *in vitro*. It was worthwhile to note that Ecn exhibited relatively less toxicity in human normal cells. Xenograft tumor model of BC cells further confirmed that Ecn significantly suppressed BC development *in vivo*. Mechanistically, we clarified that Ecn might exert its anti-tumor actions against BC cells at least partly by activating the p38 signaling pathway and inactivating the Wnt/β-catenin pathway. Moreover, we validated that Ecn combined with clinical chemotherapeutic drugs, cisplatin (DDP) or gemcitabine (Gem), synergistically suppressed the progression of BC cells. In total, our results strongly imply that Ecn may be a potential drug candidate for treating BC.

## Materials and methods

### Cell culture and drug preparations

T24 and EJ cells were cultured in RPMI 1640 medium, SV-HUC-1 cells in F12K medium, and HEB, HS5, and LO2 cells in DMEM supplemented with 10% FBS. All cells were incubated in a humidified atmosphere of 5% CO_2_ at 37 °C. Ecn (purity: 99.58%, Sichuan Cuiyirun Biotechnology Co., Ltd), SB239063, cisplatin, and gemcitabine were dissolved in dimethyl sulfoxide (DMSO) and stored at -80 °C.

### MTT viability assay

BC cells and human normal cells were plated into 96-well plates at a density of 2 × 10^3^ cells/well.Upon reaching 50% confluence, cells were treated with different concentrations of Ecn (0, 20, 30, 40, and 50 μM). At 24 h, 48 h, and 72 h after treatment, 10 μL MTT (methyl thiazole tetrazolium) solution was added to each well and co-incubated at 37 °C under dark conditions for another 4 h. Afterwards, the form azan crystals formed were dissolved with 100 μL DMSO, and the absorbance at 492 nm was measured using a multifunctional enzyme labeling instrument.

### Crystal violet staining

BC cells were seeded into 24-well plates at a density of 5 × 10^4^ cells/well. After adhesion, cells were treated with different concentrations of Ecn (0, 20, 30, 40, and 50 μM). At 24 h, 48 h, and 72 h after treatment, cells were fixed with 4% paraformaldehyde and then stained with 0.5% crystal violet. Next, crystal violet was solubilized in 200 μL of 10% glacial acetic acid at room temperature and the absorbance at 595 nm was read with a multifunctional enzyme labeling instrument.

### Colony formation assay

BC cells were inoculated into 6-well plates at a density of 1 × 10^3^ cells/well and then cultured with different concentrations of Ecn (0, 20, 30, 40, and 50 μM) for 7 days. Finally, colonies were immobilized with 4% paraformaldehyde and stained with crystal violet, and the number of colonies formed on the plates was counted.

### Hoechst 33258 assay

BC cells were seeded into 24-well plates at a density of 5 × 10^4^ cells/well. When cell confluence reached 50%, different concentrations of Ecn (0, 20, 30, 40, and 50 μM) were added to the wells. After 24 h, the culture medium was removed, and cells were stained with Hoechst 33258 dye solution. Apoptotic cells were observed and three visual fields were randomly photographed under a fluorescence microscope.

### Flow cytometry analysis

BC cells and human normal cells were seeded into 6-cm cell culture dishes. When reaching 50% confluence, cells were treated with different concentrations of Ecn (0, 20, 30, 40, and 50 μM). At 24 h after Ecn treatment, cells were collected and re-suspended by washing three times with PBS. Next, cells were stained with Annexin V-FITC/PI solution and then subjected to flow cytometry for analyzing cell apoptosis. For cell cycle determination, cells were fixed with 75% alcohol, stained with PI solution, and then analyzed by flow cytometry.

### Wound healing assay

BC cells were inoculated into 6-well plates and grown until fully confluent. Then, the cell monolayer was scraped by a 10-μL sterile pipette tip to create a wound, and the cell culture medium was substituted with fresh RPMI 1640 medium containing different concentrations of Ecn (0, 20, 30, 40, and 50 μM). At 12 h and 24 h after Ecn treatment, the wound was captured with a microscope, and the width was measured with Image J software.

### Transwell migration assay and Matrigel-coated Transwell invasion assay

For migration assay, a total of 2.5 × 10^4^ BC cells in 400 μL serum-free RPMI 1640 medium were seeded into the upper chamber, whereas the lower chamber was filled with medium containing different concentrations of Ecn (0, 20, 30, 40, and 50 μM). After 24-h incubation, non-migrated cells on the upside of the upper chamber were removed with cotton swabs. Migrated cells on the outside of the upper chamber were immobilized by paraformaldehyde fixation, stained with crystal violet, and photographed under an inverted microscope. For the invasion assay, the Matrigel solution was mixed with the culture medium in a ratio of 1:6 and then spread evenly on the upper chamber, and all subsequent steps were the same as the migration assay described above.

### Western blot analysis

BC cells were digested with trypsin, collected, and lysed in IP lysis buffer containing protease inhibitors after being treated with different concentrations of Ecn (0, 20, 30, and 40 μM) for 24 h. Protein concentrations in lysates were determined using a bicinchoninic acid assay. Then, protein samples were separated on 10% SDS-PAGE gels and transferred onto polyvinylidene fluoride membranes. Blotted membranes were blocked by bovine serum albumin and then sequentially incubated with corresponding primary antibodies at 4 °C for 16 h, with secondary antibodies at 37 °C for 2 h. Finally, protein bands were visualized with an ECL kit and photographed by the ChemiDoc XRS imaging system.

### Network pharmacology

The secondary structure of Ecn was available at PubChem (https://pubchem.ncbi.nlm.nih.gov/). Based on the structure, the Ecn-related targets were obtained from the PharmMapper database (http://lilab-ecust.cn/pharmmapper/). The BC-related targets were collected from the GeneCards database (www.genecards.org/) using “bladder cancer” as the keyword. Afterward, the intersection between Ecn-related and BC-related targets was identified by the online Venn diagram tool. The protein–protein interaction (PPI) network of the obtained intersection was constructed with the STRING database and visualized with the Cytoscape software.

### Establishment of xenograft tumor model

T24 cells (4 × 10^7^ cells) suspended in 80 μL sterile PBS were subcutaneously injected into the right flank of female BALB/c nude mice (4 weeks old) to establish the xenograft tumor model. Ecn (0, 20, 40, and 60 mg/kg) or 0.5% sodium carboxymethyl cellulose (CMC) was administered by oral gavage once every two days for 21 days. Body weight and tumor volume were measured and recorded at the same time. At the experimental endpoint, all mice were sacrificed and tumor tissues were harvested.

### Hematoxylin and eosin (H&E) staining

Tumor tissues were fixed with 4% paraformaldehyde, embedded in paraffin, and cut into 5-μm sections. Tissue sections were dewaxed and then subjected to H&E staining according to the standard histopathological protocol. At least three areas of each sample were randomly photographed under an optical microscope.

### Immunohistochemistry (IHC) analysis

Tumor tissues were fixed, embedded in paraffin, and sectioned at 5-μm thickness. The sections were dewaxed and then rehydrated with 100%, 90%, 80%, and 70% ethanol. Subsequently, sections were incubated with primary antibodies at 4 °C overnight. After incubation, sections were washed 3 times with PBS and incubated with secondary antibodies coupled with horseradish peroxidase. The target proteins were visualized using the DAB kit and the images were observed under an optical microscope.

### Assessment of drug combination by Jin's formula

Jin's formula is Q = E_a + b_/(E_a_ + E_b_−E_a_ × E_b_), where E_a_ is the inhibition rate of drug A, E_b_ is the inhibition rate of drug B. The classification is as follows: *Q* < 0.85 indicates the antagonistic effect; 0.85 ≤ *Q* < 1.15 indicates the additive effect; *Q* ≥ 1.15 indicates the synergistic effect.

### Statistical analysis

All experiments were performed in triplicate. Data were presented as means ± standard error of the mean and processed by GraphPad Prism 9.0 software (GraphPad Software, La Jolla, CA, USA). The comparisons between the two groups were performed using a two-tailed Student's *t*-test. The comparisons among three or more groups were assessed by One-way ANOVA. A *P*-value <0.05 was considered statistically significant.

## Results

### Ecn inhibits the proliferation and induces G2/M arrest of BC cells

**Figure 1:**
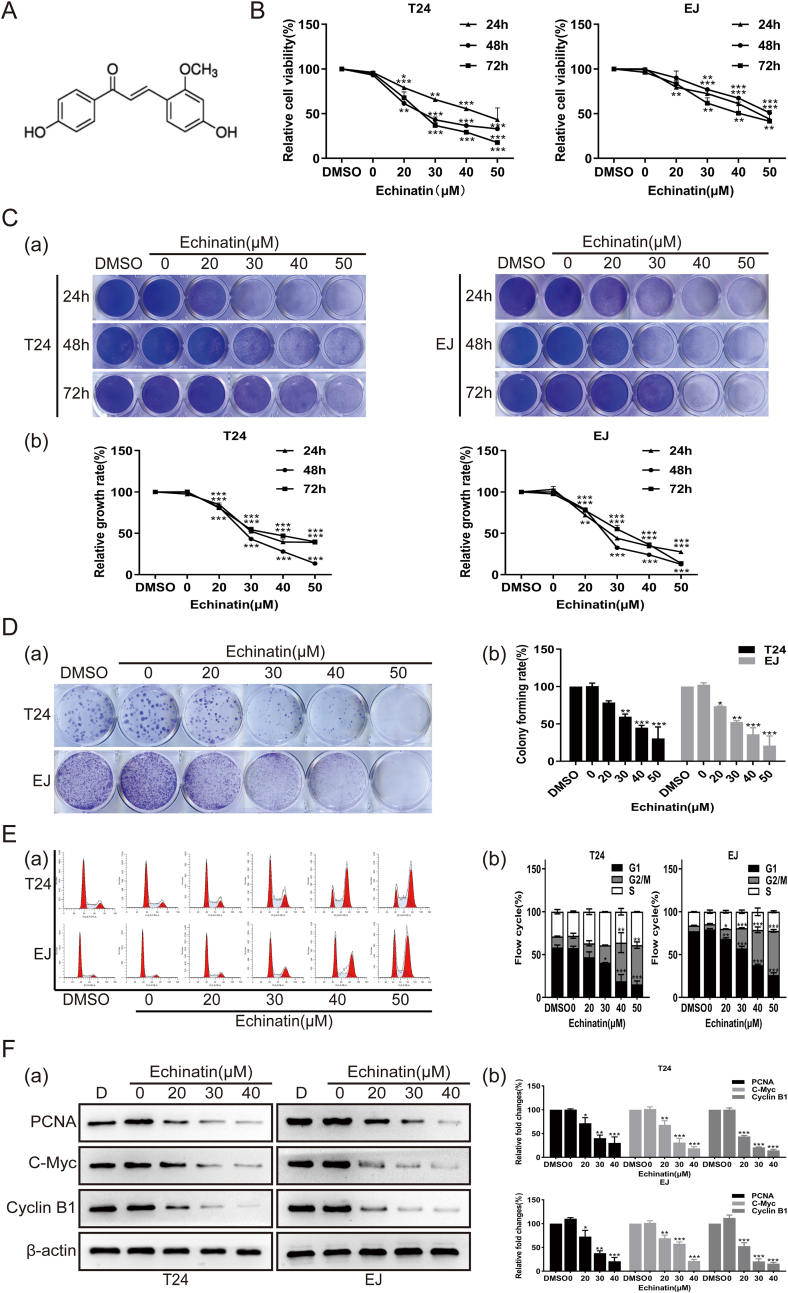
Ecn inhibits BC cell proliferation *in vitro*. **(A)** The chemical structure of Ecn. **(B)** The effect of Ecn on the viability of T24 and EJ cells (MTT).**(C)**The effect of Ecn on the viability of T24 and EJ cells (crystal violet staining). Panel (a): representative images of crystal violet staining. Panel (b): quantification of crystal violet staining. **(D)** The effect of Ecn on the colony-forming ability of T24 and EJ cells (colony formation assay). Panel (a): representative images of colony formation assay. Panel (b): quantification of colony formation assay. **(E)** The effect of Ecn on the cell cycle progression of T24 and EJ cells (flow cytometry). Panel (a): representative images of flow cytometry. Panel (b): quantification of flow cytometry. **(F)** The effect of Ecn on levels of PCNA, c-Myc, and cyclin B1 of T24 and EJ cells (Western blot). Panel (a): representative images of Western blot. Panel (b): quantification of Western blot. ^∗^*P* < 0.05, ^∗∗^*P* < 0.01, ^∗∗∗^*P* < 0.001, *vs*. the 0 μM Ecn group.

The chemical structure of Ecn was presented in Figure 1A. First, we evaluated the effects of Ecn on the proliferation of BC cells. We conducted MTT assays and found that Ecn significantly inhibited the viability of BC cells mostly in a dose-dependent manner (Fig. 1B). The IC_50_ values of Ecnin T24 cells and EJ cells were 43.85 μM and 47.21 μM, respectively. The inhibitory effect of Ecn on the viability of BC cells was further verified by crystal violet staining (Fig. 1C). In addition, we found that Ecn treatment considerably diminished the *in vitro* colony formation capacity of BC cells, resulting in a visible reduction in the number of colonies formed on the plates (Fig. 1D). Further, we conducted Western blot and demonstrated that the protein levels of PCNA (proliferating cell nuclear antigen) and c-Myc (v-mycmyelocytomatosis viral oncogene homolog), two well-recognized predictors of cell proliferation, were sharply reduced in Ecn-treated BC cells compared to untreated cells (Fig. 1F). Considering that cell proliferation is closely linked to cell cycle, we, therefore, performed flow cytometry analysis to monitor the cell cycle progression of BC cells. As shown in Figure 1E, we observed prominent G2/M phase arrest of cell cycle distribution in Ecn-treated BC cells, represented by an increased proportion of G2/M phase cells whereas a decreased proportion of G1 phase cells. Correspondingly, the result of Western blot showed that the protein level of Cyclin B1, which is a master Cyclin promoting G2/M phase transition, was down-regulated by Ecn remarkably (Fig. 1F). Collectively, these results imply that Ecn may inhibit the proliferation and trigger G2/M arrest of BC cells.

### Ecn induces the apoptosis of BC cells

**Figure 2:**
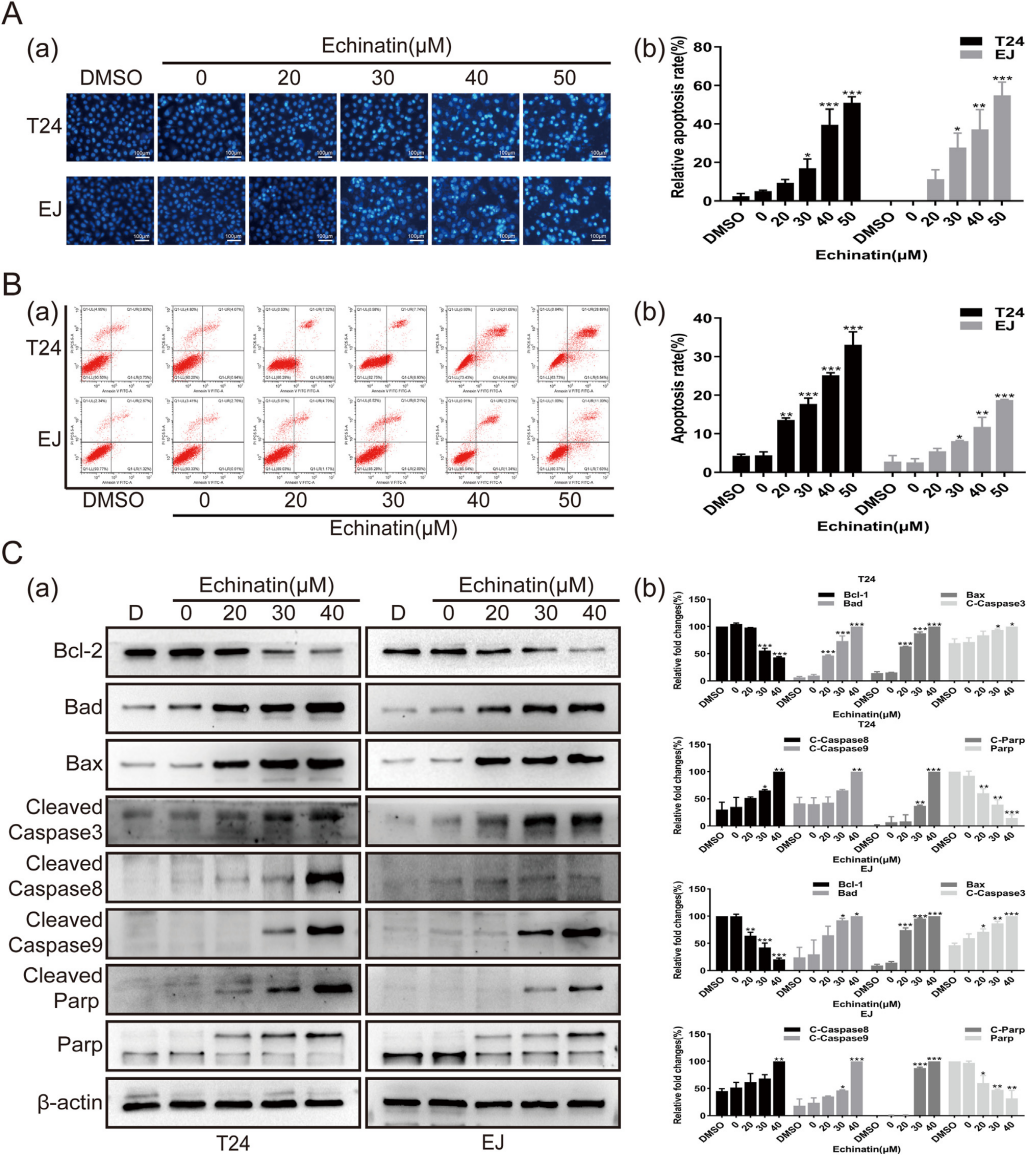
Ecn promotes apoptosis of BC cells. **(A)** The effect of Ecn on apoptosis of T24 and EJ cells (Hoechst 33258 staining, 100×). Panel (a): representative images of Hoechst 33258 staining. Panel (b): quantification of Hoechst 33258 staining. **(B)** The effect of Ecn on apoptosis of T24 and EJ cells (flow cytometry). Panel (a): representative images of flow cytometry. Panel (b): quantification of flow cytometry. **(C)** The effect of Ecn on levels of apoptosis-related proteins of T24 and EJ cells (Western blot). Panel (a): representative images of Western blot. Panel (b): quantification of Western blot. ^∗^*P* < 0.05, ^∗∗^*P* < 0.01, ^∗∗∗^*P* < 0.001, *vs*. the 0 μM Ecn group.

As the activation of cell apoptosis is one of the leading causes of cell death and proliferation inhibition, we therefore determined whether Ecn affected apoptosis in BC cells. Using Hoechst 33258 staining, we observed that compared with untreated cells, Ecn-treated BC cells showed visible morphological features typical of apoptosis such as bright blue-fluorescent condensed/fragmented nuclei (Fig. 2A). Consistently, flow cytometry analysis also revealed that the apoptosis rate of BC cells was noticeably increased by Ecn treatment (Fig. 2B). Next, we analyzed the changes of apoptosis-related molecules upon Ecn treatment by employing Western blot. We found that the levels of cleaved-caspase3, cleaved-Parp, cleaved-caspase 8, and cleaved-caspase 9, which are all excellent bio-markers reflecting the degree of apoptosis activation, were elevated significantly after Ecn treatment. Moreover, we demonstrated a remarkable elevation of apoptosis-promoting factors Bad and Bax, whereas a marked reduction of apoptosis-suppressing molecule Bcl-2 in Ecn-treated BC cells (Fig. 2C). Thus, these results suggest that Ecn may trigger apoptosis in BC cells.

### Ecn has no apparent cytotoxicity on human normal cells

**Figure S1:**
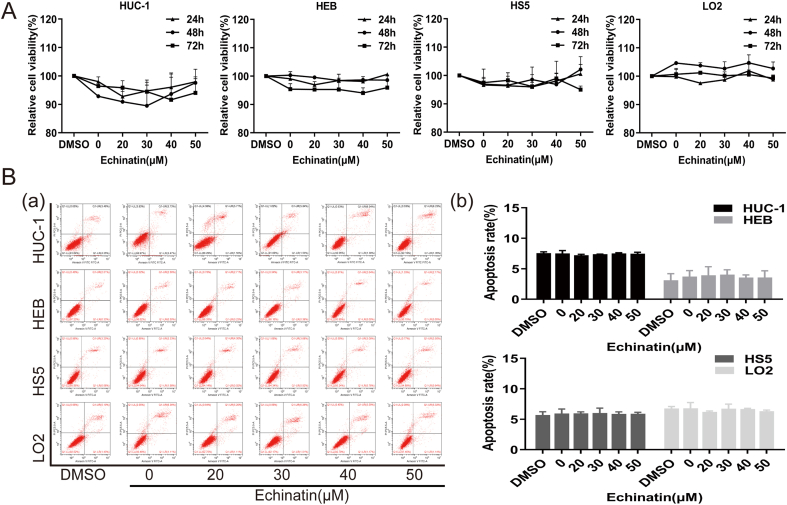
Ecn has no significant toxic effect on normal cells. **(A)** The effect of Ecn on the proliferation of SV-HUC-1, HEB, HS5, and LO2 cells (MTT). **(B)** The effect of Ecn on apoptosis of SV-HUC-1, HEB, HS5, and LO2 cells (flow cytometry). Panel (a): representative images of flow cytometry. Panel (b): quantification of flow cytometry. ∗*P* < 0.05, ∗∗*P* < 0.01, ∗∗∗*P* < 0.001, *vs*. the 0 μMEcn group.

Next, we assessed the safety of Ecn in human normal cells. We treated SV-HUC-1, HEB, HS5, and LO2 cells with Ecn at the same doses that effectively caused *in vitro* inhibitory effects on BC cells. We then validated by MTT assay that the viability of these normal cells was barely affected by Ecn with the same concentration used in BC cells (Fig. S1A). Moreover, we found by flow cytometry that Ecn almost had no visible effect on the apoptosis of BC cells (Fig. S1B). The above results collectively indicate the preliminary safety of Ecn because it has selective cytotoxicity toward BC cells, but not against normal cells.

### Ecn suppresses the migration and invasion of BC cells

**Figure 3:**
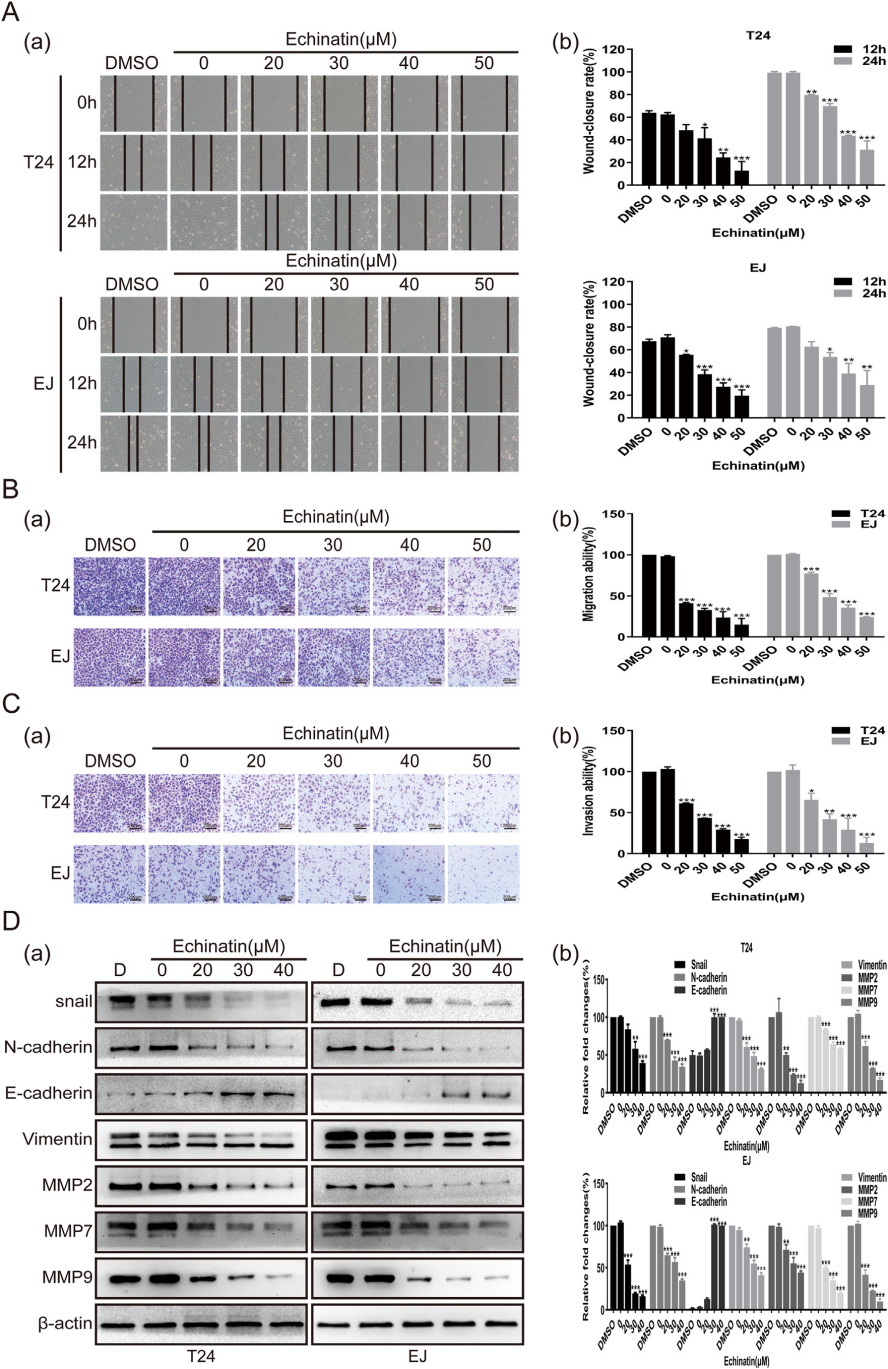
Ecn suppresses the migration and invasion of BC cells. **(A)** The effect of Ecn on the migration of T24 and EJ cells (wound healing assay, 200×). Panel (a): representative images of wound healing assay. Panel (b): quantification of wound healing assay. **(B)** The effect of Ecn on the migration of T24 and EJ cells (Transwellassay, 200×). Panel (a): representative images of Transwellassay. Panel (b): quantification of Transwellassay. **(C)** The effect of Ecn on the invasion of T24 and EJ cells (Maltrigel-coated Transwellassay, 200×). Panel (a): representative images of Maltrigel-coated Transwellassay. Panel (b): quantification of Maltrigel-coated Transwellassay. **(D)** The effect of Ecn on levels of EMT markers and MMPs of T24 and EJ cells (Western blot). Panel (a): representative images of Western blot. Panel (b): quantification of Western blot. ^∗^*P* < 0.05, ^∗∗^*P* < 0.01, ^∗∗∗^*P* < 0.001, *vs*. the 0 μM Ecn group.

BC often displays a high tendency to metastasize, leading to a poor prognosis. We, therefore, sought to explore whether Ecn had any inhibitory influence on the migration and invasion of BC cells. By employing wound healing assays, we demonstrated that the wound healing ability of Ecn-treated BC cells was apparently attenuated, indicating an inhibitory effect of Ecn on the migration of BC cells (Fig. 3A). Using Transwellmigration assay, we further confirmed this inhibitory effect with the results that Ecn significantly reduced the number of migrating BC cells passing through the upper chamber (Fig. 3B). Besides, we disclosed by Transwellinvasion assay that the number of invasive BC cells penetrating cross the Matrigel-coated membrane in Ecn-treatment group was much lower than that of the control group (Fig. 3C). Thus, these findings suggest that Ecn may restrain the migration and invasion of BC cells. Next, we investigated whether Ecn influenced epithelial–mesenchymal transition (EMT) which is one of the key determinants of migration and invasion. Using Western blot, we verified a reversed EMT phenotype in Ecn-treated BC cells, as evidenced by significant down-regulation of the EMT-promoting factors snail, N-cadherin, and vimentin and up-regulation of the EMT-suppressing factor E-cadherin. Moreover, the results of Western blot also showed that Ecn effectively reduced the protein levels of MMP2, MMP7, and MMP9, which are essential executioners catalyzing the degradation of extracellular matrix (ECM) in BC cells (Fig. 3D). Taken together, these data indicate that Ecn may attenuate the migration and invasion abilities of BC cells via reversing the EMT process and inhibiting MMPs.

### Ecn exerts inhibitory effects on BC cells at least partly through regulating p38 and Wnt/β-catenin signaling pathways

**Figure 4:**
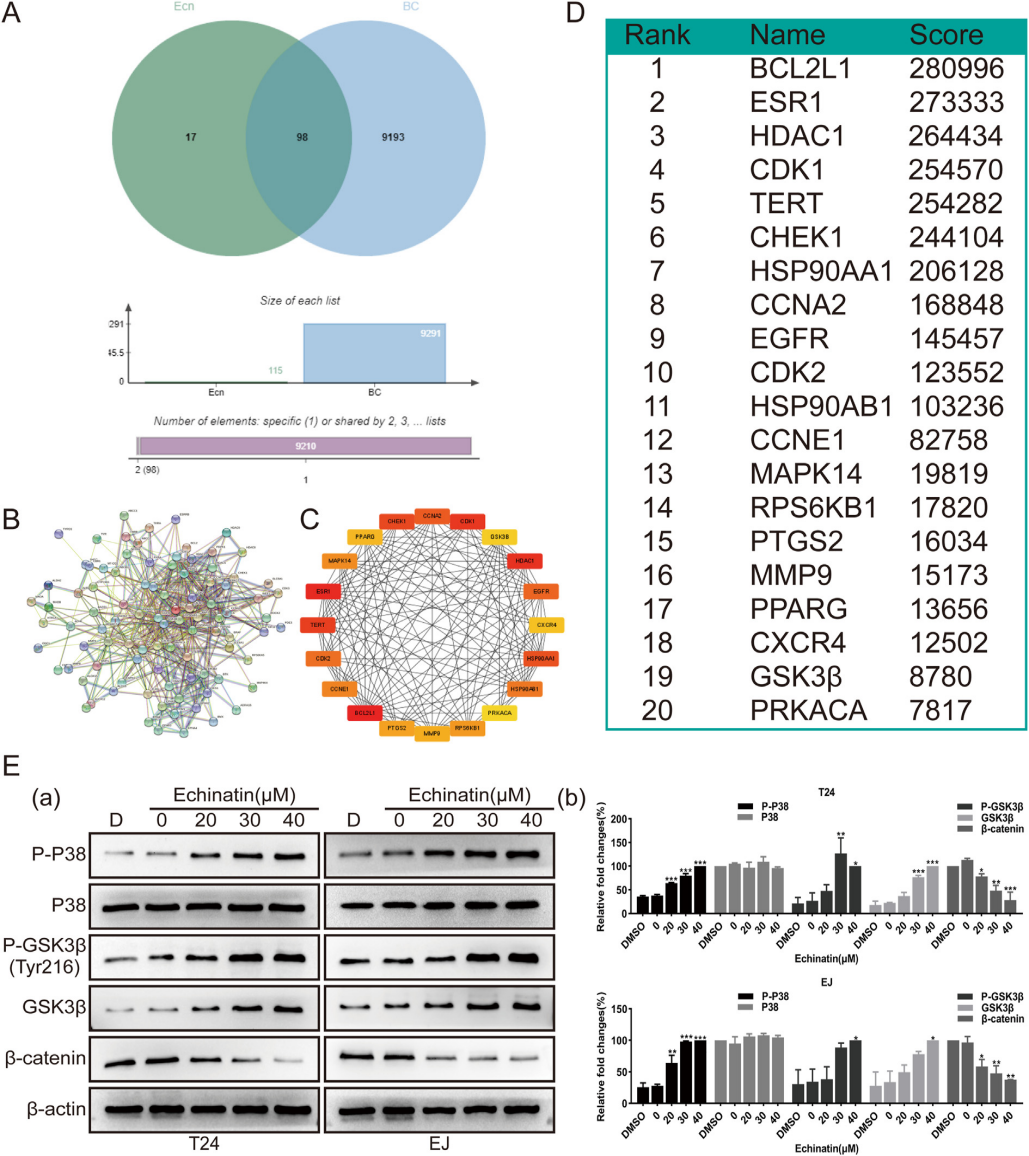
Ecn activates p38 signaling pathway and suppresses the Wnt/β-catenin signaling pathway of BC cells. **(A)** The depicted targets of Ecn against BC (Venn diagram). **(B, C)** The PPI network depicted targets of Ecn against BC. **(D)** The brief information of the top 20 hub protein targets in the PPI network ranked by MCC value. **(E)** The effect of Ecn on p38 and Wnt/β-catenin signaling pathways of T24 and EJ cells (Western blot). Panel (a): representative images of Western blot. Panel (b): quantification of Western blot. ^∗^*P* < 0.05, ^∗∗^*P* < 0.01, ^∗∗∗^*P* < 0.001, *vs*. the 0 μM Ecn group.

Next, we dissected the possible mechanism through which Ecn inhibited BC cells. First, we performed network pharmacological analysis to predict the potential targets of Ecn against BC. Overall, 115 unique Ecn-related targets were obtained from the PharmMapper database, and 9291 potent BC-related targets were collected from the GeneCards database. After taking the intersection of Ecn-related and BC-related targets by Venn diagram analysis, we finally identified 98 intersection targets (Fig. 4A), namely the targets of Ecn action on BC. We then constructed a PPI network of these intersection targets to depict the direct and indirect regulatory interactions (Fig. 4B). According to the PPI network, we screened out the top 20 hub proteins and ranked them by MCC value (Fig. 4C). Among the 20 hub proteins (Fig. 4D), MAPK14 (mitogen-activated protein kinase 14) and GSK3β (glycogen synthase kinase 3 beta) in particular aroused our interest since these two molecules are both important kinases that determine the activation of p38 and Wnt/β-catenin signaling pathways, which are closely related to the occurrence and development of tumors. Therefore, we investigated whether p38and GSK3β in BC cells were affected by Ecn. Using Western blot assays, we found that Ecn promoted p38 phosphorylation without visibly altering the total p38 protein in BC cells (Fig. 4E), implying the activation of the p38 signaling pathway. As the total GSK3β protein and the active form of GSK3β (tyrosine 216 phosphorylation, p-GSK3β (Tyr216)) were both elevated upon Ecn treatment (Fig. 4E), we speculated that Ecn might inhibit Wnt/β-catenin signaling pathway. This speculation was further evidenced by Western blot that Ecn decreased the level of β-catenin protein in BC cells (Fig. 4E). In total, these results reveal that Ecn may activate the p38 signaling pathway and inactivate Wnt/β-catenin signaling pathways in BC cells.

**Figure 5:**
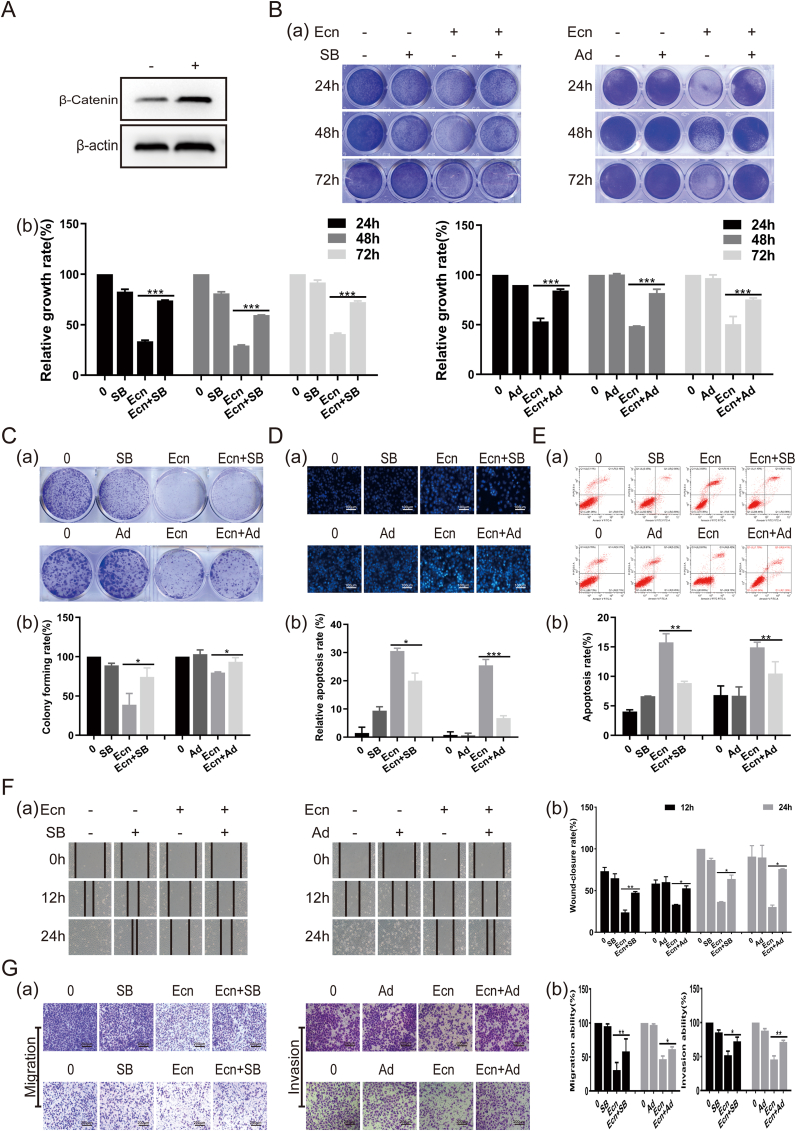
Activation of p38 signaling pathway and inactivation of the Wnt/β-catenin signaling pathway mediate the inhibitory effects of Ecn of BC cells. **(A)** Over-expression of β-catenin in T24 cells by adenovirus Ad-β-catenin (Western blot). **(B)** The effect of Ecn on the viability of T24 cells in the presence of SB239063 or Ad-β-catenin (crystal violet staining). Panel (a): representative images of crystal violet staining. Panel (b): quantification of crystal violet staining. **(C)** The effect of Ecn on the colony formation ability of T24 cells in the presence of SB239063 or Ad-β-catenin (colony formation assay). Panel (a): representative images of colony formation assay. Panel (b): quantification of colony formation assay. **(D)** The effect of Ecn on apoptosis of T24 cells in the presence of SB239063 or Ad-β-catenin (Hoechst 33258 staining, 100×). Panel (a): representative images of Hoechst 33258 staining. Panel (b): quantification of Hoechst 33258 staining. **(E)** The effect of Ecn on apoptosis of T24 cells in the presence of SB239063 or Ad-β-catenin (flow cytometry). Panel (a): representative images of flow cytometry. Panel (b): quantification of flow cytometry. **(F)** The effect of Ecn on the migration of T24 cells in the presence of SB239063 or Ad-β-catenin (wound healing assay, 200×). Panel (a): representative images of wound healing assay. Panel (b): quantification of wound healing assay. **(G)** The effect of Ecn on the migration and invasion of T24 cells in the presence of SB239063 or Ad-β-catenin (Transwellmigration and invasion assay, 200×). Panel (a): representative images of Transwellmigration and invasion assay. Panel (b): quantification of Transwellmigration and invasion assay. ^∗^*P* < 0.05, ^∗∗^*P* < 0.01, ^∗∗∗^*P* < 0.001, *vs*. the single Ecn group.

We then determined whether the activation of the p38 signaling pathway and the inactivation of the Wnt/β-catenin signaling pathway might, at least in part, mediate Ecn-induced inhibition of BC cells. We treated T24 BC cells with 20 μM of Ecn in the presence of the p38inhibitor SB239063 (15 μM) or adenovirus over-expressing β-catenin (Ad-β-catenin). Then, we discovered that Ecn-induced inhibition of proliferation, migration, and invasion, and Ecn-triggered apoptosis was partially reversed by SB239063 and Ad-β-catenin (Fig. 5). Overall, these results suggest that Ecn may exert anti-tumor effects on BC cells at least partially via p38 and Wnt/β-catenin signaling pathways.

### Ecn inhibits *in vivo* xenograft tumor development of BC cells

**Figure 6:**
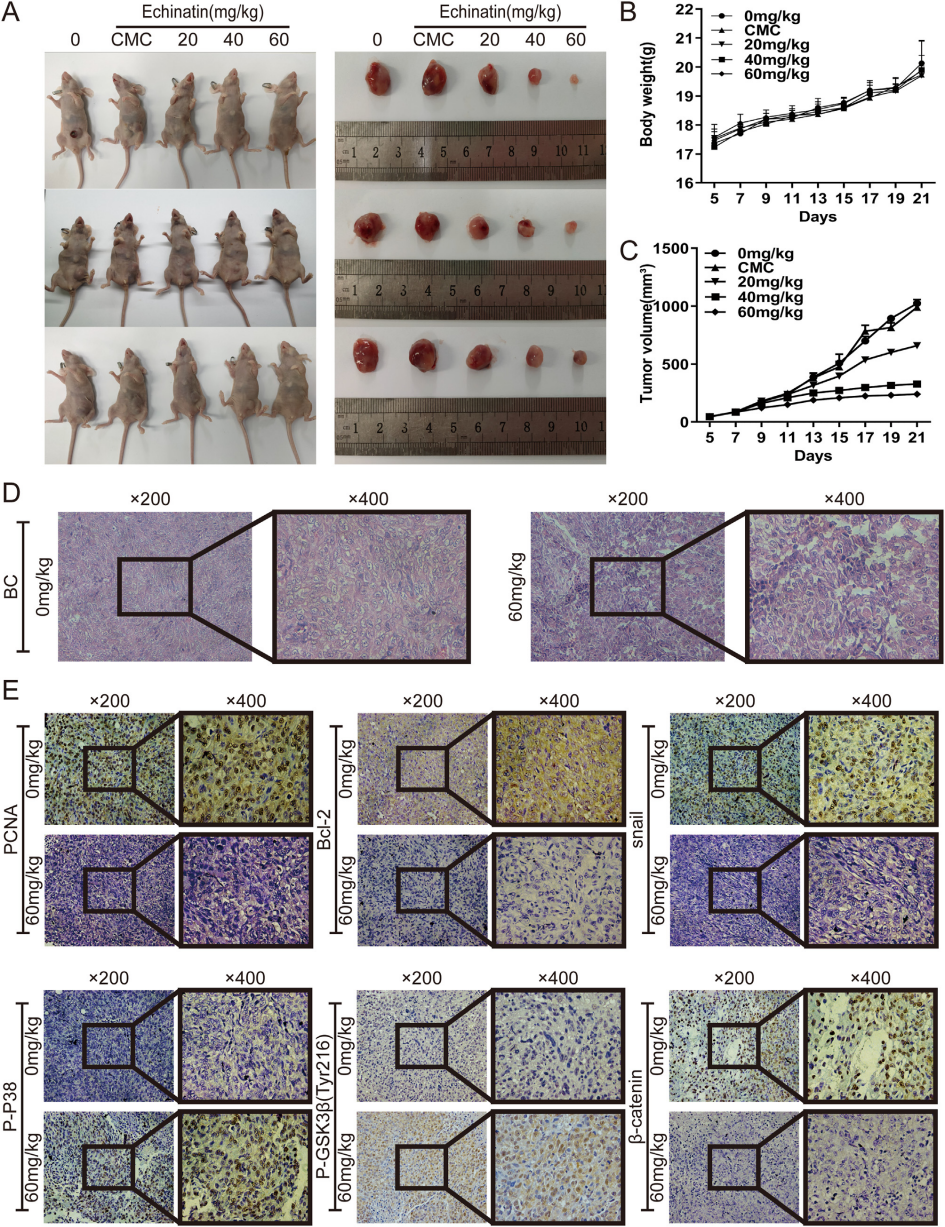
Ecn suppresses the xenograft tumor development of BC cells *in vivo*. **(A)** Representative images of nude mice and subcutaneous tumors. **(B)** The body weight of nude mice. **(C)** The volume of the xenograft tumor of BC cells. **(D)** Representative H&E staining of BC tumor tissues. **(E)** The levels of PCNA, Bcl-2, snail, P–P38, p-GSK3β, and β-catenin of BC tumor tissues (IHC). ^∗^*P* < 0.05, ^∗∗^*P* < 0.01, ^∗∗∗^*P* < 0.001, *vs*. the 0 mg/kg Ecn group.

As Ecn exerted a considerable inhibitory effect on BC cells *in vitro*, we, therefore, established a subcutaneous xenograft tumor model of BC cells further to confirm this suppressive effect of Ecn*in vivo.* Compared with the control group, the Ecn treatment group showed a substantial reduction in the tumor volume (Fig. 6A, C) without obvious loss of body weight (Fig. 6A, B). Further, using H&E staining, we observed apparent malignant phenotypes of cells such as deeply stained nuclei and unbalanced nuclear/cytoplasmic ratio in the control group. However, Ecn effectively decreased these malignant phenotypes in BC cells (Fig. 6D). These results imply that Ecn may retard the xenograft tumor progression of BC cells *in vivo*. On the other hand, IHC analysis demonstrated that Ecn decreased the levels of PCNA, Bcl-2, snail, and β-catenin while increasing p-p38 and p-GSK3β(Tyr216) (Fig. 6E), which is consistent with Western blot results. In conclusion, these data suggest that Ecn may suppress the growth and metastasis of BC cells *in vivo*.

### Ecn combined with cisplatin or gemcitabine produces synergistic inhibitory effects on BC cells

**Figure 7:**
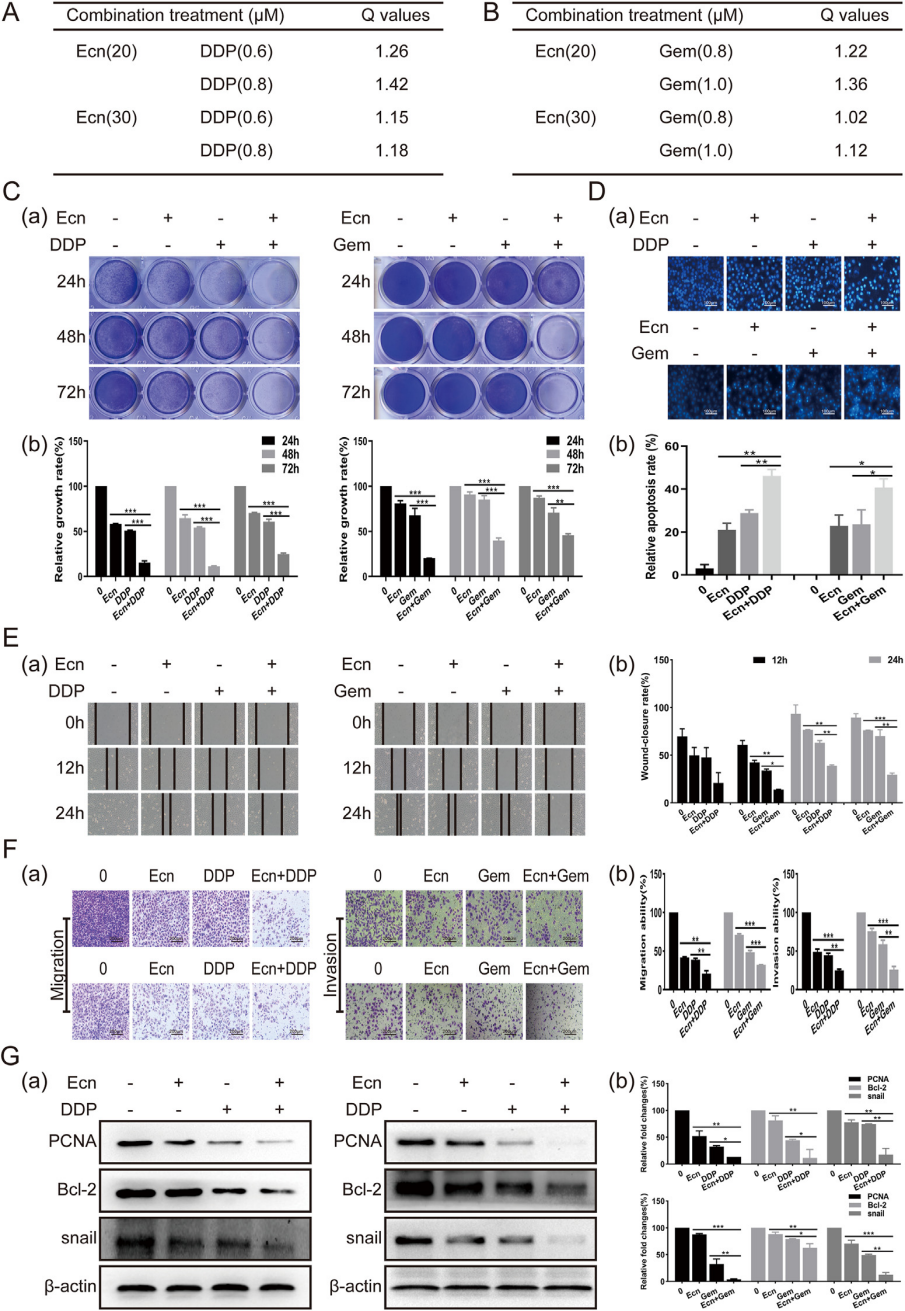
Ecn combined with DPP or Gem synergistically inhibits the BC cell progression. **(A, B)** The effects of Ecn combined with DPP or Gem on T24 cells (Jin's formula analysis). **(C)** The effect of Ecn combined with DPP or Gem on the viability of T24 cells (crystal violet staining). Panel (a): representative images of crystal violet staining. Panel (b): quantification of crystal violet staining. **(D)** The effect of Ecn combined with DPP or Gem on apoptosis of T24 cells (Hoechst 33258 staining, 100×). Panel (a): representative images of Hoechst 33258 staining. Panel (b): quantification of Hoechst 33258 staining. **(E)** The effect of Ecn combined with DPP or Gem on the migration of T24 cells (wound healing assay, 200×). Panel (a): representative images of wound healing assay. Panel (b): quantification of wound healing assay. **(F)** The effect of Ecn combined with DPP or Gem on the migration of T24 cells (Transwellmigration and invasion assay, 200×). Panel (a): representative images of Transwellmigration and invasion assay. Panel (b): quantification of Transwellmigration and invasion assay. **(G)** The effect of Ecn combined with DPP or Gem on levels of PCNA, Bcl-2, and snail of T24 cells (Western blot). Panel (a): representative images of Western blot. Panel (b): quantification of Western blot. ^∗^*P* < 0.05, ^∗∗^*P* < 0.01, ^∗∗∗^*P* < 0.001, *vs*. the single drug group.

At present, the combination of drugs is an effective strategy for anti-tumor therapy in clinical practice because drug combinations might have synergistic tumoricidal effects. Thus, we thought to confirm whether Ecn in combination with cisplatin (DDP) or gemcitabine (Gem) had any synergistic inhibitory effect on BC cells. From the results of Jin's formula analysis, we found that Ecn (20 μM) combined with DDP (0.8 μM) or Gem (1.0 μM) had a better synergistic inhibitory effect on the proliferation of T24 BC cells (Fig. 7A–C). We also validated that Ecn combined with DDP or Gem attenuated the migration and invasion while promoting apoptosis of T24 cells in a synergistic manner (Fig. 7D–F). Consistently, the combination groups demonstrated a more pronounced reduction of PCNA, Bcl-2, and snail protein levels compared with the monotherapy groups (Fig. 7G). To sum up, these results support that the combination of Ecn with DDP or Gem may synergistically inhibit the progression of BC cells.

## Discussion

Even though chemotherapy is frequently implemented in BC treatment, its efficacy is often limited by tumor metastasis, chemo-resistance, long-term side effects, and nonspecific cytotoxicity toward normal cells.^26^^,^^27^ Therefore, it is still an urgent issue to develop novel anti-BC drugs with high efficiency and low toxicity. Natural products isolated from Chinese herbs are considered rich sources for new drug development, and increasing evidence has highlighted the therapeutic potential in tumor treatment.^28^ Ecn is a natural flavonoid that can be mainly extracted from the traditional Chinese medicine licorice.^21^ Although Ecn exhibits multiple pharmacological effects, its anti-tumor effect has only recently been preliminarily verified in esophageal, lung, and colorectal cancer.^23^, ^24^, ^25^ In this current study, we demonstrated that Ecn significantly inhibited the growth and metastasis of human BC cells possibly through p38 and Wnt/β-catenin signaling pathways *in vitro* and *in vivo*. Also, it is important to emphasize that the combination of Ecn with DDP or Gem produced synergistic inhibitory effects on BC cells.

Aberrant proliferation is one of the distinctive characteristics of tumor cells.^29^^,^^30^ Therefore, the capacity to inhibit the proliferation of tumor cells is viewed as an evaluation index for anti-tumor drugs. In the present study, we demonstrated that Ecn considerably inhibited cell viability and colony formation ability of BC cells. PCNA is an important DNA replication determining molecular that directly participates in the initiation of proliferation by interacting with DNA polymerase delta.^31^ It is highly and specifically expressed in actively proliferating normal cells and infinitely replicating tumor cells.^32^ c-Myc is a nuclear transcription factor encoded by the proto-oncogene c-Myc and is often activated and/or over-expressed in various tumors. c-Myc endows tumor cells with infinite proliferative ability and functions as an essential mediator in multiple proliferative pathways.^33^^,^^34^ Therefore, PCNA and c-Myc are both widely recognized as excellent indicators reflecting cell proliferation accurately. In this present study, we found that Ecn decreased the levels of PCNA and c-Myc, inhibited cell viability and clone formation *in vitro*, and restrained xenograft tumor growth of BC cells *in vivo*. This evidence intensively supports that Ecn has an excellent anti-proliferation effect on BC cells. The proliferation of eukaryotic cells heavily depends on an orderly cell cycle process composed of four distinct phases (G1, S, G2, and M), and cell cycle disruption will lead to the impairment of cell proliferation.^35^ In fact, most of the existing anti-BC drugs available clinically, such as cisplatin and gemcitabine, can induce cell cycle arrest at specific phases to limit cell proliferation, thus eventually causing cell death.^36^^,^^37^ Cell cycle is a finely-coordinated process precisely controlled by numerous regulatory factors, among which cyclins are probably the most critical. Cyclins are a class of protein family consisting of several members that are specifically expressed in different cell cycle phases.^38^ As a key member of cyclins, cyclin B1 is exclusively expressed in the G2 and M phases and is necessary for the completion of the G2/M phase transition by interacting with CDK1.^39^ Therefore, down-regulation of cyclin B1 will consequently enforce the G2/M phase arrest of the cell cycle. Previous studies have consistently reported that Ecn induced G2/M arrest in esophageal cancer cells, lung cancer cells, and colorectal cancer cells.^23^, ^24^, ^25^ Interestingly, we observed similar phenomena in Ecn-treated BC cells, evidenced by the accumulation of cells in the G2/M phase and the significant reduction of cyclin B1. Therefore, these results indicate that Ecn may have the activity of specifically inducing G2/M phase arrest in different tumor cells, including BC cells, and highlight its potential as a cell cycle blocker for tumor therapy.

Apoptosis induction is the common mechanism of most existing anti-tumor drugs and is considered one of the essential criteria for developing new anti-tumor drugs.^40^ In this study, we revealed typical apoptotic phenotypes in Ecn-treated BC cells, suggesting that Ecn may induce apoptosis of BC cells. Combined with the results of previous studies that Ecn markedly induced apoptosis in esophageal cancer cells, lung cancer cells, and colorectal cancer cells, we propose that Ecn is probably a potent apoptosis inducer in various tumors. It should be emphasized that the reported mechanisms of Ecn-induced apoptosis are presumably diverse in different tumor cells. For instance, in esophageal cancer cells, Ecn induced apoptosis by inactivating AKT/mTOR signaling pathway,^23^ while in colorectal cancer cells, it promoted apoptosis by activating JNK and p38 MAPK signaling pathways.^25^ Therefore, further research is required to clarify the specific mechanism underlying Ecn-induced apoptosis in BC cells. BC cells are especially prone to metastasis. In this study, we also demonstrated that Ecn significantly attenuated the migration and invasion of BC cells by reversing the EMT process and inhibiting MMPs. All these findings provide clear evidence that Ecn has robust anti-proliferative, pro-apoptotic, and anti-metastatic effects on human BC cells. More meaningfully, Ecn may have acceptable safety because Ecn in the same concentration range almost hardly interferes with the viability and apoptosis of several types of normal cells.

Using network pharmacology analysis, we finally got several possible targets for Ecn acting on BC cells. Among the top 20 targets, BCL2L1, MMP9, and CDK1 are important molecules involved in apoptosis, metastasis, and cell cycle, respectively.^41^, ^42^, ^43^, ^44^ These findings further indicate that Ecn may play an anti-BC role by influencing the above-mentioned three aspects. Although all targets warrant deeper investigation, MAPK14 (also called p38) and GSK3β are of particular interest to us because these two are essential kinases that directly determine the activity of p38 and Wnt/β-catenin signaling pathways. As a member of the MAPK family, the role of p38 in tumors appears to be ambiguous and complex. Some studies have suggested that p38 activation is beneficial for tumor development and inhibition of p38 may cause tumor repression,^45^^,^^46^ whereas others presented opposite results.^47^^,^^48^ Nonetheless, p38 provides a plausible mechanism for certain natural products with anti-tumor potential. For instance, Chen et al reported that protodioscin inhibited bladder cancer cell migration and growth, and promoted apoptosis by activating the p38 signaling pathway.^49^ A study by Dang et al demonstrated that significant inactivation of the p38 signaling pathway was responsible for the kaempferol-mediated cell proliferation inhibition.^50^ Here, using Western blot assays, we found that Ecn increased p38 phosphorylation in BC cells, indicating that the p38 signaling pathway is activated by Ecn. Moreover, the p38 inhibitor SB239063 effectively neutralized the inhibitory effect of Ecn on BC cells. These findings are very similar to those of Ecn in colorectal cancer cells, that is, Ecn inhibited oxaliplatin-sensitive and -resistant CRCs by activating the p38 signaling pathway, while the p38 inhibitor weakened the inhibitory effects of Ecn.^25^ We, therefore, conclude that Ecn may exert anti-BC effects, at least partly, by activating the p38 signaling pathway.

In the present study, GSK3β was another target predicted by our network pharmacology analysis. GSK3β is a critical determinant in the Wnt/β-catenin pathway. It can catalyze the phosphorylation of β-catenin and subsequently enhance the ubiquitinated degradation of β-catenin, ultimately leading to the supersession of the Wnt/β-catenin pathway.^51^ The catalytic activity of GSK3β for β-catenin degradation heavily depends on its phosphorylation status, and phosphorylation at the tyrosine 216 site will increase the catalytic activity.^52^ Abnormal activation of the Wnt/β-catenin signaling pathway can result in uncontrolled proliferation and malignant transformation of cells.^53^^,^^54^ Wnt/β-catenin signaling pathway is often aberrantly activated in BC and contributes to the development and progression of the disease.^55^ Drugs that can inhibit the Wnt/β-Catenin signaling pathway may have excellent anti-BC potential. For example, the carbonic anhydrase inhibitor acetazolamide was able to inhibit urinary bladder cancer via suppression of β-catenin signaling.^56^ Curcumin was reported to reverse chronic tobacco-elicited EMT and acquisition of stemness properties through inhibition of Wnt/β-catenin in BC cells.^57^ Here, we verified that Ecn promoted the phosphorylation of GSK3β at tyrosine 216, implying an increased GSK3β catalytic activity. Accordingly, the β-catenin protein was reduced in Ecn-treated BC cells. These results suggest that Ecn may activate GSK3β, thus leading to the inhibition ofthe Wnt/β-catenin signaling pathway. Moreover, we demonstrated that over-expression of β-catenin neutralized the inhibitory effects of Ecn on BC cells. It thus suggests that the inactivation of the Wnt/β-catenin signaling pathway by GSK3β activation may also be a mechanism for the anti-BC effect of Ecn.

Combination chemotherapy refers to the combined administration of two or more chemotherapeutic drugs. It can improve the anti-tumor effect, and reduce the dosage and lower the side effects of drugs through the synergistic effect of different chemotherapy drugs.^58^ According to European Association of Urology guidelines, combination chemotherapy regimens including GC (gemcitabine plus cisplatin) and MVAC (methotrexate, vinblastine, and doxorubicin plus cisplatin) have been recommended as the standard first-line treatment for BC patients.^59^ The combinations of natural products and clinically used anti-BC drugs might have potential synergistic anti-BC effects as well. For example, curcumin and its derivatives can act synergistically with both cisplatin and gemcitabine to inhibit BC cells.^60^ In addition, shikonin combined with cisplatin can exhibit significantly greater killing effects on BC cells than a single drug.^61^ In the current study, we validated that Ecn combined with cisplatin or gemcitabine produced synergistic inhibitory effects on BC cells. Thus, Ecn may have excellent potential in improving both cisplatin- and gemcitabine-based chemotherapy for bladder cancer.

Overall, our study not only suggests that Ecn may serve as an efficacious therapeutic agent for the treatment of BC but also provides a potential strategy to enhance the anti-BC effect of clinically used drugs by combining them with Ecn.

## Ethics declaration

All animal experiments were approved by the Animal Ethics Committee of Chongqing Medical University.

## Author contributions

Y.J.L. conceived and supervised the study. X.X.W. and J.L.L. performed the experiments and drafted the manuscript. T.J.X. and P.Q.L provided the standard protocols of experiments. C.H.X. and R.Y.H. helped to perform the experiments. L.L.Z and P.L.X performed the data analyses. B.H.W.J and Q.S.L performed data preparation and statistics. J.X.L and Y.J.L. reviewed the paper. All authors read and approved the final version of this manuscript.

## Conflict of interests

The authors declare that they have no competing interests.

## Funding

This work was supported by the 10.13039/501100001809National Natural Science Foundation of China (No. 81874001) and the Program for Youth Innovation in Future Medicine of Chongqing Medical University (China) (No. W0086).
